# Real-world patient outcomes with Blinatumomab and Inotuzumab in adult relapsed/refractory B-cell acute lymphoblastic leukemia: a retrospective analysis from two Romanian oncology centers

**DOI:** 10.3389/fphar.2025.1684382

**Published:** 2025-12-10

**Authors:** Ion Antohe, Amalia Titieanu, Vlad Andrei Cianga, Cosmin Minciună, Cătălin Dănăilă, Alina Daniela Tănase, Iuliu Ivanov, Loredana Dragoş, Mihaela Zlei, Mihaela Mențel, Ciprian Tomuleasa, Anamaria Bancos, Manuela Ciocoiu, Angela Dăscălescu

**Affiliations:** 1 Grigore T. Popa University of Medicine and Pharmacy, Iaşi, Romania; 2 Bone Marrow Transplant Department, Regional Oncology Institute, Iaşi, Romania; 3 Hematology Department, Regional Oncology Institute, Iaşi, Romania; 4 Bone Marrow Transplant Department, Fundeni Clinical Institute, Bucharest, Romania; 5 Molecular Diagnostic Department, Regional Oncology Institute, Iaşi, Romania; 6 Flow Cytometry Department, Regional Oncology Institute, Iaşi, Romania; 7 Department of Personalized Medicine and Rare Diseases, Medfuture Institute for Biomedical Research – Department of Hematology, Iuliu Hațieganu University of Medicine and Pharmacy, Cluj-Napoca, Romania; 8 Department of Hematology, Ion Chiricuță Cancer Center, Cluj Napoca, Romania; 9 Physiopathology Department, Faculty of Medicine, “Grigore T. Popa” University of Medicine and Pharmacy, Iaşi, Romania

**Keywords:** B-cell acute lymphoblastic leukemia, immunotherapy, CAR-T cells, Blinatumomab, Inotuzumab

## Abstract

**Introduction:**

The management of adult relapsed/refractory B-cell acute lymphoblastic leukemia (R/R B-ALL) remains a significant challenge, with patients presenting particularly poor historical outcomes. Novel immunotherapies, such as Blinatumomab and Inotuzumab, have revived the therapeutic landscape of R/R B-ALL, but their optimal sequencing, toxicity management, and post-transplant integration remain unclear, particularly in real-world, resource-limited settings.

**Methods:**

We conducted a retrospective analysis of 33 adult patients with R/R B-ALL treated between 2019 and 2023 at two major oncology centers in Romania.

**Results:**

Blinatumomab and Inotuzumab achieved complete remission rates of 47.6% and 90%, respectively, with measurable residual disease negativity in over 60% of responders. Inotuzumab was more effective in early relapses and cases with high disease burden, while Blinatumomab showed benefit in later relapses and post-transplant settings. Eleven patients were successfully bridged to allogeneic stem cell transplant, and four were treated for post-transplant relapse, demonstrating the feasibility of immunotherapy in both contexts. Sequential monoclonal antibody use yielded poor survival unless used as a bridge to curative therapy.

**Conclusion:**

Our findings emphasize the need for individualized treatment selection, rational immunotherapy sequencing, and the development of predictive biomarkers to optimize outcomes and minimize toxicity. Further studies are needed to refine therapeutic algorithms and define such prognostic biomarkers, particularly in underfunded healthcare systems.

## Introduction

The management of B cell acute lymphoblastic leukemia (B-ALL) in the adult population remains especially challenging, since virtually all patients are at high risk of leukemia relapse and long-term disease-free survival (DFS) ranges from 20% to 35% (Kantarjian et al., 2000). Furthermore, relapsed/refractory (R/R) patients are faced with a paucity of treatment options and, ultimately, a dismal prognosis (Gökbuget et al., 2012; Oriol et al., 2010; Spyridonidis et al., 2012).

However, the therapeutic landscape of B-ALL has been fundamentally changed by the emergence of the novel immunotherapies: Blinatumomab, a CD3-CD19 bi-specific T cell engager, Inotuzumab, a chalicheamicin-bound anti-CD22 monoclonal antibody (mAb) and tisagenlecleucel, a CD19-specific chimeric antigen receptor modified T cell (CAR-T) therapy, all of which have induced complete remissions and even measurable residual disease (MRD) negativity in up to 80% of R/R patients (Khazal and Kebriaei, 2020). These remissions provide the patient with a narrow window of opportunity to be bridged to allogeneic stem cell transplant, which offers a chance to achieve long-term survival. Even more, a growing body of data shows that these agents have even more efficacy if used as a first-line approach (Jabbour et al., 2025; Hodder et al., 2024). Despite these promising advancements, many issues still remain unclear. How to select and, eventually, sequence the immunotherapy agent to maximize the chance of achieving a second complete remission (CR), while also minimizing unnecessary toxicity by avoiding agents unlikely to be effective? Is allogeneic stem cell transplantation (allo-SCT) still necessary after all types of immunotherapies? How to approach patients with post-alloSCT relapse? Given the many uncertainties in the field, it has become increasingly important to identify which patient subgroups are most likely to benefit from each therapy, and to establish sound principles for sequencing these novel immunotherapies in the heterogeneous population of R/R patients.

Furthermore, clinical experience in real-life settings often differs from that reported in clinical trials, and real-world data on immunotherapy in the already rare context of R/R B-ALL- particularly in the even rarer post-transplant setting-is scarce. We thus aim to present the experience of two major oncology centers from Romania with adult R/R B-ALL patients who were treated with immunotherapy and to review literature data regarding the role and sequencing strategies of immunotherapies in the pre- and post-transplant setting.

## Materials and methods

We conducted a retrospective multi-center analysis of adult patients with R/R B-ALL that were treated between 2019 and 2023 with Blinatumomab, Inotuzumab, or both, at the Regional Oncology Institute from Iaşi and the “Ion Chiricuță” Oncoloy Institute from Cluj-Napoca, Romania. This study was approved by the local ethics committees. Blinatumomab and Inotuzumab were administered following the standard dosage schedule approved by the European Medicines Agency and the Romanian Health Ministry National Protocol. Blinatumomab has been approved in Romania for R/R Philadelphia (Ph) negative B-ALL since 2018 and for Ph positive disease in 2022, while Inotuzumab can be administered for R/R B-ALL irrespectively of the Ph status since 2020.

Patients who received Blinatumomab solely for MRD eradication were excluded, in order to maintain a clinically homogeneous cohort restricted to relapsed/refractory disease.

Complete remission (CR) was defined as a bone marrow (BM) percentage of lymphoblasts below 5%, the absence of extramedullary leukemia and hematologic reconstitution (transfusion independence, absolute neutrophil count >1,000/μL, platelet count >100000/μL). CR with incomplete hematologic recovery (CRi) was defined if the first two criteria of the above are met, but without achieving hematologic recovery.

MRD was assessed by 8 color multiparameter flow cytometry using a BD FACSLyric™ Clinical Flow Cytometry System. Samples were processed according to the EuroFlow standard operating protocol, using a Bulk Lysis technique (https://euroflow.org/protocols/) for the enrichment of rare target cell subsets. The MRD assays achieved the sensitivity of up to 0.001% (1-in-10^5^) with the acquisition of up to 5 million cells/tube. Markers used in the MRD panels were selected according to the EuroFlow consortium recommendations (in terms of the clone, fluorochrome combination, saturating titer) and were combined in 1/2 tubes that always included CD10, CD19, CD20, CD34, CD45, CD38 (for the identification of hematogones, mature B cells and plasma cells). Additionally, for the measurement of residual malignant clones, depending on the aberrancy detected at diagnosis, the following markers were used: CD13, CD22, CD33, CD58, CD66c, CD123, TdT.

Cytokine release syndrome (CRS) and neurotoxicity to Blinatumomab was defined in accordance to the NCI CTCAE v 5.0 criteria. Tumor lysis syndrome (TLS) was defined according to the Cairo-Bishop grading system (Cairo and Bishop, 2004).

### Statistical analysis

The statistical analysis was performed using the IBM SPSS Statistics 21.0 Software. Mean and range were used to describe continuous variables. Fisher’s exact test was utilized to investigate associations between categorical variables. The Chi square test was used to analyze the associations between different variables. A *P* value <0.05 was considered statistically significant. Overall survival (OS) and disease-free survival (DFS) were estimated using the Kaplan-Meier method. The log rank test was used for comparisons of Kaplan-Meier curves. OS was defined as the time from the initiation of the first monoclonal antibody to death from any cause. DFS was calculated separately for each monoclonal antibody, from treatment start until relapse or death of any cause. Given the limited sample size, we did not perform multivariate analyses, as the statistical power would have been insufficient. Therefore, we conducted only univariate analyses.

## Results

**FIGURE 1:**
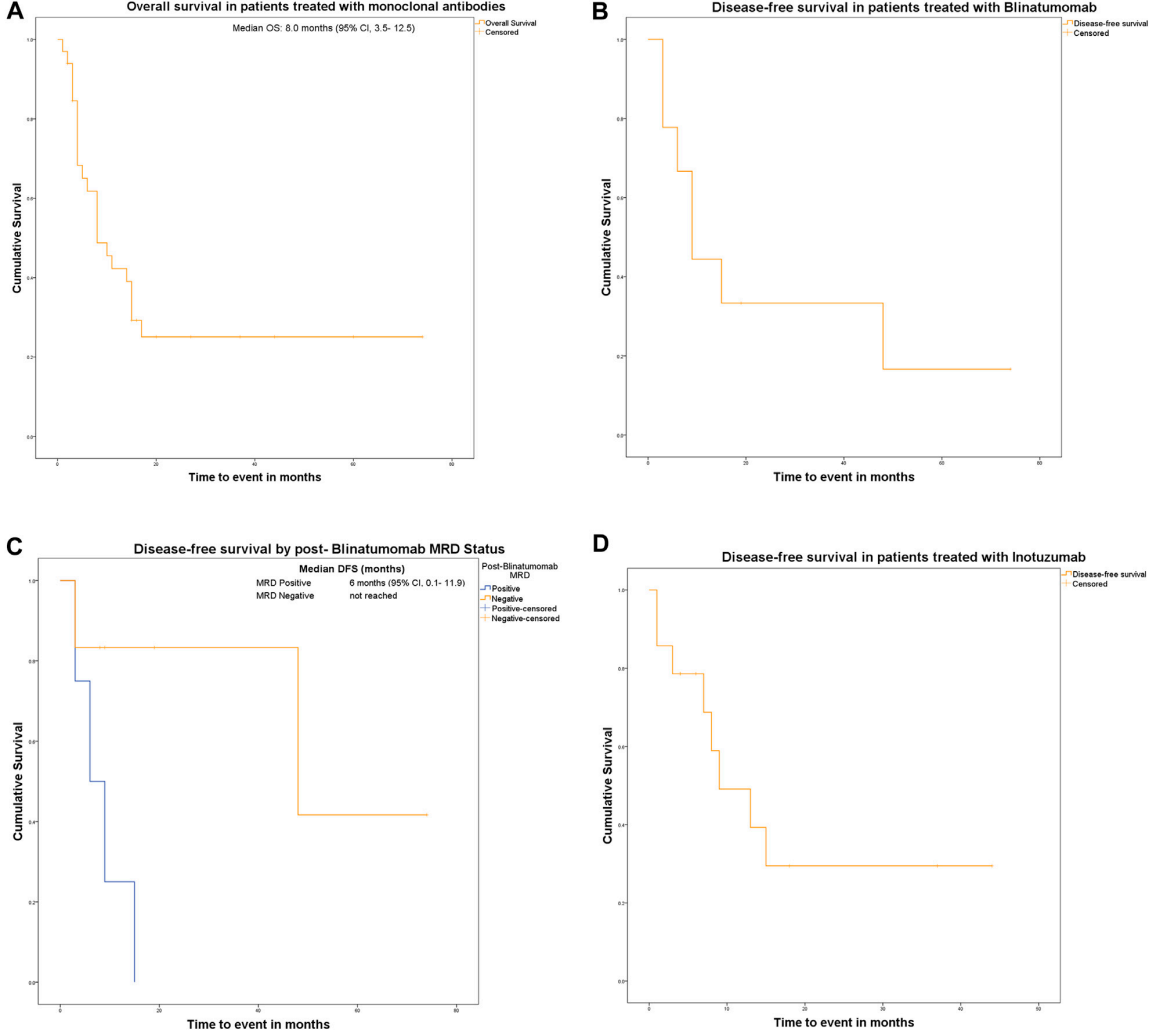
**(A)** Kaplan–Meier overall survival (OS) curve for all patients treated with monoclonal antibodies. The estimated median OS was 8.0 months (95% CI, 3.5−12.5). **(B)** Kaplan–Meier disease-free survival (DFS) curve for patients treated with Blinatumomab. The estimated median DFS was 15.0 months (95% CI, 0.2−29.8). **(C)** Kaplan–Meier disease-free survival (DFS) curves for patients treated with Blinatumomab, stratified by post-treatment MRD status. Patients with MRD positivity had a median DFS of 6.0 months (95% CI, 0.1−11.9), whereas the median DFS for MRD-negative patients was not reached (P = 0.037, log-rank test). **(D)** Kaplan–Meier disease-free survival (DFS) curve for patients treated with Inotuzumab. The estimated median DFS was 8.0 months (95% CI, 4.1−11.9).

Our study group comprised 33 patients with R/R B-ALL treated between 2019 and 2023. 84.8% (n = 29) of cases were Ph negative. 24.2% (n = 8) of patients had primary refractory B-ALL. 63.6% of patients (n = 21) received Blinatumomab and 60.6% (n = 20) were treated with Inotuzumab. Both immunotherapies were administered sequentially in 24.2% (n = 8). The median duration of follow-up was 20 months. The estimated median OS was 8 months (95% CI, 3.5–12.5 months) (Figure 1A). We successively analyzed patients treated with Blinatumomab, patients treated with Inotuzumab and, finally, three patient subgroups that pose particular challenges to the practitioner and deserve special attention: 1) patients sequentially treated with both monoclonals; 2) patients who were bridged to transplant, and 3) patients with posttransplant relapse. The baseline characteristics of patients are shown in Table 1.

**TABLE 1:** Baseline patient characteristics.

Patient characteristics				
	Total	Blinatumomab	Inotuzumab	Both
Sex (M/F), n (%)	23/10 (69.7/30.3)	14/7 (42.4/21.2)	14/6 (42.4/18.1)	5/3 (15.1/9)
Age, median (range)	40 (18–61)	42 (18–67)	41 (18–71)	56 (30–65)
CNS involvement, n (%)	3 (9)	1 (3)	2 (6)	1 (3)
Laboratory data				
• WBC x 10^9^/L, median (range)	3.35 (1.1–15.6)	4 (1.1–15.6)	2.700 (1.2–11.1)	5.75 (2.7–8.6)
• BM blast percentage, median (range)	60 (5–95)	39 (5–95)	80 (6–95)	36 (17–60)
Philadelphia status, n (%)	6 (23)	0	4 (15.3)	2 (7.7)
CD19 expression, median % (range)	100 (50–100)	100 (50–100)	-	90 (80–100)
CD22 expression, median % (range)	50 (10–100)	-	70 (30–100)	45 (10–70)
Prior allo-SCT, n (%)	4 (15.3)	2 (7.7)	2 (7.7)	0

allo-SCT, allogeneic stem cell transplant; BM, bone marrow; CNS, central nervous system; WBC, white blood cell count.

### Blinatumomab

Blinatumomab was administered to 21 patients (63.6%). Detailed patient and pre-treatment characteristics are summarized in Table 1. The overall response rate (ORR) to Blinatumomab was 47.6% (CR = 70%, CRi = 30%), with MRD negativity achieved in 66% of responders. 12 and 24-month DFS were 50% and 42%, respectively. The estimated median DFS was 15.0 months (95% CI, 0.2–29.8) (Figure 1B). For MRD positive patients, the median DFS was 6 months (95% CI, 0.1–11.9) (Figure 1C). In the MRD negative group, the median DFS was not reached, as fewer than half of the patients had relapsed at the time of analysis; however, the Kaplan–Meier model generated an estimated median DFS of 48 months (95% CI, 0–112.4), which should be interpreted with caution given the small number of events (only two patients relapsed in the MRD negative group). The therapies that preceded Blinatumomab administration are shown in Supplementary Table S1. Out of the three patients receiving purine-based analogue chemotherapy before Blinatumomab, only one achieved MRD negative CR. Other factors, such as central nervous system (CNS) infiltration, BM blast percentage, the Ph status and the use of Blinatumomab as first or later salvage did not impact OS and DFS. The safety data is being shown in Table 2.

**TABLE 2:** The toxicities of immunotherapies.

Immunotherapy parameters	Blinatumomab N = 21	Inotuzumab N = 20
First/Later salvage, n (%)	15 (71.4)/6 (28.6)	10 (50)/10 (50)
Primary refractory ALL	5 (23.8)	4 (25)
Philadelphia positive	0 (0)	2 (10)
Post-allo-SCT relapse, n (%)	2 (9.5)	2 (10)
Adverse effects, n (%)	9 (42.8)	14 (70)
Hematologic toxicity	5 (23.8)	16 (80)
Grade I-II	3 (14.2)	12 (60)
Grade III-IV	2 (9.5)	4 (20)
Transaminitis	0	3 (15)
SOS	0	2 (10)
CRS	4 (19)	0
TLS	2 (9.5)	0
Neurotoxicity	1 (4.7)	0

ALL, acute lymphoblastic leukemia; Allo-SCT, allogeneic stem cell transplant; CRS, cytokine release syndrome; SOS, sinusoidal obstruction syndrome; TLS, tumor lysis syndrome.

### Inotuzumab

Inotuzumab was administered to 20 patients (60.6%), with a median number of two cycles. A summary of patient characteristics is provided in Table 1. The ORR to Inotuzumab was 90% (n = 18) (CR = 72.3%, CRi = 27.7%). MRD negativity was achieved in 83.3% of cases (n = 15). The ORR in Ph positive cases was 80%, with all patients achieving MRD negativity. The estimated median DFS was reached at 8 months (95% CI, 4.1–11.9) (1-year DFS: 26.3%) (Figure 1D). The estimated DFS was 36% at 10 months and 24% at 15 months. Three patients were MRD-positive, with no significant influence on DFS (*P* = 0.445, log-rank test; data not shown).

There was no significant statistical difference in response rates when comparing the use of Inotuzumab as first and later salvage, although a trend towards statistical significance could be evidenced (First salvage ORR: 100%; later salvage ORR: 71.4%) (*P* = 0.042, Chi-square test, *P* = 0.1, Fisher’s exact test; data not shown). CNS infiltration and the BM blast percentage did not impact response rates to Inotuzumab. Primary refractoriness to Inotuzumab was documented in two cases, in whom it was used as second salvage option. The adverse effects to Inotuzumab are shown in Table 2.

### Patients treated sequentially with both monoclonal antibodies

Both treatments were administered sequentially in eight patients (24.2%). Four patients received Blinatumomab as the first mAb. The median duration between the two mAbs was 6 months. The CR rate for the first mAb used was 87.5% (7 cases). When analyzing the response rates separately, 100% of patients responded to Inotuzumab and 75% to Blinatumomab. When patients were treated with the second immunotherapy, the CR rate was 37.5% (3 cases) (Inotuzumab response rate 50%, Blinatumomab response rate 25%). Only one patient was refractory to both immunotherapies. Two patients were successfully bridged to transplant with Inotuzumab and one of the transplanted patients was successfully bridged to CAR-T with Blinatumomab upon relapse. No patient was successfully bridged to alloSCT with Blinatumomab. Overall, the median OS for the patients treated with the first immunotherapy was 6 months. On the other hand, patients treated with the second monoclonal had a survival rate of 12.5% at 6 months.

### Patients with CNS involvement

CNS infiltration could be evidenced before immunotherapy in three patients (9%). In two cases, it was synchronous with BM relapse and was eradicated with intrathecal (IT) chemotherapy followed by high-dose cytarabine before Blinatumomab. The third case presented with late (day +185), post-alloSCT, isolated CNS relapse and was refractory to IT chemotherapy and Methotrexate. The patient responded to cerebrospinal irradiation and was then treated with Inotuzumab upon medullary relapse. Overall, the presence of CNS infiltration did not impact OS (*p* = 0.3, log-rank test) and DFS (*p* = 0.7, log-rank test) (data not shown) in our study group.

### Outcomes of patients who received allo-SCT following immunotherapy

Eleven patients underwent allo-SCT after immunotherapy, of which eight (72.7%) were successfully bridged with Inotuzumab and two (18%) with Blinatumomab. One patient (9%) proceeded to allo-SCT with progressive leukemia after failure of both mAbs. Ten patients were transplanted in MRD negative CR. Transplant-specific clinical variables are presented in Table 3. No patient received total body irradiation.

**TABLE 3:** Transplant specific variables of patients treated with Blinatumomab and Inotuzumab.

Transplant characteristics	Inotuzumab (n = 9)	Blinatumomab (n = 2)
Disease status at SCT CR, MRD negative, n	8	2
Prior immunotherapy	Blinatumomab- 1 case	No
Conditioning regimen		
MAC, n RIC, n	7 2	0 2
Dual-alkylator conditioning, n	4	0
GVHD prophylaxis		
TACRO/MMF, n PTCy, n CSA/Metothrexat, n	8 4 1	1 0 1
Acute GVHD	1	1
Chronic GVHD	0	1
SOS	2	0

CR, complete remission; CSA, Cyclosporin A; FLAG, fludarabine, Cytarabine, Granulocyte colony stimulating factor; MAC, myeloablative conditioning; MRD, measurable residual disease, N/A, not applicable; PTCy, post-transplant Cyclophosphamide; RIC, reduced intensity conditioning; SCT, stem cell transplant; SOS, sinusoidal obstruction syndrome.

Posttransplant DFS was 27.2% at 1 year (Median reached at 6 months). Median posttransplant OS was reached at 7 months. Two-year OS was 36.3%. When compared to non-transplanted patients, patients who underwent allo-SCT showed a survival benefit, without achieving statistical significance (1-year OS: 54.5% vs. 31.8% (*p* = 0.2, log-rank test), 2-year OS 27.2% vs. 9% (*p* = 0.2, log-rank test). Moderate sinusoidal obstruction syndrome (SOS) was noticed in two Inotuzumab-treated cases (22.2%). The duration from Inotuzumab to allo-SCT in patients who developed SOS was 28 and 31 days, respectively. One of the patients developing SOS received a dual alkylator regimen containing Thiothepa. Both patients achieved recovery from SOS after 11 and 15 days, respectively. Neither patient required treatment with defibrotide, as both cases were moderate in severity and recovered with supportive care alone.”

### Outcomes of patients who received immunotherapy for post-transplant relapse

Two patients were treated with Inotuzumab and two with Blinatumomab for post-allo-SCT relapse. Blinatumomab patients had Ph negative B-ALL and late post-alloSCT relapses (at 35 and 48 months, respectively). On the other hand, Inotuzumab patients had Ph positive B-ALL and earlier relapses, at 6 and 9 months, respectively. Both Blinatumomab treated patients also received donor lymphocyte infusions (DLI), while Inotuzumab was administered in association with tyrosine kinase inhibitors.

Blinatumomab cases received the drug as first salvage and achieved MRD negative CR after two cycles. The time from Blinatumomab to DLI was 32 and 60 days, respectively. The DLI doses were escalated as follows: 5 × 10^6^/kg, 14 × 10^6^/kg, 71 × 10^6^/kg for the first case and 1 × 10^7^/kg, 5 × 10^7^/kg, 0.5 × 10^8^/kg for the second. None of the patients received graft versus host disease (GVHD) prophylaxis. One patient developed acute, steroid refractory GVHD at day +26 following the last DLI dose, which responded to Ruxolitinib. This patient had no GVHD antecedents and received the first DLI dose before the last Blinatumomab cycle. In the second patient, DLI was administered after Blinatumomab. Both patients achieved and maintained MRD negative CR and, in the second case, the patient remains alive, GVHD-free at 48 months from DLI.

Both patients treated with Inotuzumab and tyrosine kinase inhibitors (TKI) achieved CR, MRD negative after two cycles. DFS was 42 and 9 months, respectively.

## Discussion

Despite its high initial remission rate, adult B-ALL relapses in approximately 60% of patients, which results in an extremely poor prognosis. This landscape has been significantly changed by the introduction of the novel mABs, superior to chemotherapy in terms of tolerability, response and bridging to allo-SCT, which should then provide long-term disease control. However, choosing one mAb over the other is challenging, as each has distinct efficacy and toxicity profiles that differentially impact subsequent treatment options, such as allo-SCT or CAR-T cell therapies.

Real world clinical experience, especially in developing countries, often differs slightly from that of clinical trials or from more advanced healthcare systems. It is highly likely that various particularities of the Romanian healthcare system and patients, including a higher incidence of comorbidities and hospital infections, low availability of blood products, disparities in patient education, challenges in accessing public health services (particularly for individuals residing in rural areas), deficiencies in the primary care system, and comparatively lower levels of public health expenditure compared to other European Union member countries, impact the prognosis of leukemia in these patients (Vladescu et al., 2016; Petre et al., 2023). Our study, conducted at two prominent oncology centers from Romania, aims therefore to present the outcomes of R/R B-ALL patients treated with one or, sequentially, with both monoclonal antibodies in the real-life setting of a developing country.

Despite the impressive results of the novel immunotherapies in the setting of R/R B-ALL, many issues remain to be clarified about the selection and sequencing of these therapies and the allo-SCT procedure: How to select immunotherapy for the first relapse? Is further consolidation in the form of allo-SCT still necessary if MRD negativity is achieved? How to sequence immunotherapies in the setting of multiple relapses, especially if allo-SCT or CAR-T cells are later planned? Finally, how do we choose and even sequence immunotherapy in the setting of post-allo-SCT relapse and could these agents promote the emergence or even relapse of GHVD? These complex therapeutic decisions, which will be explored in the following sections, must be approached on a case-by-case basis, as they are influenced by multiple interdependent factors, including the regulatory approval status of immunotherapies, their efficacy and toxicity profiles, CNS infiltration, the leukemic burden, the intent for subsequent allogeneic transplantation, the logistical considerations related to drug availability and administration, the clinical context of post-transplant relapse, and the potential impact on the risk of GVHD.

### The approval status of the immunotherapies

In the real-life setting, the first factor to impact the clinician’s choice is the approval status of these drugs by the national healthcare insurance system. In Romania, as well as in the rest of Europe, Blinatumomab is the only agent to be reimbursed in both the adult and pediatric population. Inotuzumab is only approved in adult patients with R/R disease, irrespectively of the Ph status. As of 2022, tisagenlecleucel is the only approved CAR-T product for patients younger than 25 years.

### Logistics of administration

Of the two immunotherapy drugs, Inotuzumab is the more easily administered as a short intravenous infusion administered weekly in an outpatient setting. Thus, Inotuzumab is preferable in patients who are not compliant to the prolonged hospitalization, the need for intravenous access, the continuous infusion and the frequent bag changes required by Blinatumomab therapy.

### Effectiveness

The landmark TOWER trial compared Blinatumomab to standard of care chemotherapy in 405 adult patients with R/R Ph negative B-ALL. Significantly higher overall response rates (44% vs. 25%), longer duration of remission (7.3 vs. 4.4 months), improved OS (7.7 vs. 4 months) and higher MRD negativity rates among responders (78% vs. 48%) were noted for Blinatumomab. Approximately one-third of patients from each treatment arm had undergone previous HCT and 24% of patients from each arm proceeded to HCT. Furthermore, multiple studies have confirmed that HCT significantly improves OS and DFS in patients who achieve CR, particularly MRD negative, following Blinatumomab treatment (Algeri et al., 2025; Yoon et al., 2019; Sayyed et al., 2024).

The INO-VATE ALL clinical trial included 326 adult patients with R/R B-ALL treated with Inotuzumab. The CR and MRD negativity rates (80.7% and 78.4%, respectively) were significantly higher in the Inotuzumab arm when compared to standard of care chemotherapy. An improved prognosis was noted if patients were successfully bridged to allo-SCT, especially if they were MRD negative (Jabbour et al., 2020). Median OS and DFS were significantly improved in the Inotuzumab arm (7.7 vs. 6.7 months and 5 vs. 1.8 months, respectively).

Based on published trials, it would appear that the efficacy of Blinatumomab is lower than that reported for Inotuzumab (44% compared with 58%–80%) (Curran and O’Brien, 2020). Different groups have attempted to indirectly compare the data from the TOWER and INO-VATE trials, but results have been conflicting (Proskorovsky et al., 2019; Proskorovsky et al., 2020; Song et al., 2020; Song et al., 2019). Moreover, real-world data showed comparable rates of CR and CRi for the two drugs (Badar et al., 2020a; Badar et al., 2020b).

In our experience, Inotuzumab had a higher overall response rate when compared to Blinatumomab (90% vs. 47.6%). However, the mechanism of action of Blinatumomab itself, which relies heavily on the activation and expansion of endogenous T cells, justifies a lower response rate, since the both T cell fitness and the immune evasion strategies of leukemic cells are highly polymorphic across different patients. Inotuzumab, on the other hand, relies on the direct delivery of a cytotoxic compound and is thus less prone to immune escape-driven resistance. Conversely, therapies that induce T-cell lymphopenia and functional impairment, such as purine analogues, are expected to lower response rates when administered prior to Blinatumomab. This was confirmed in our study group, as only 33% of patients pre-treated with Fludarabine responded to Blinatumomab.

In responders, Inotuzumab achieved slightly higher rates of MRD negativity compared to Blinatumomab. However, patients treated with Blinatumomab who achieved MRD negativity had a clearly superior prognosis when compared to the MRD positives. By contrast, our findings suggest that MRD status did not significantly influence survival outcomes in patients treated with Inotuzumab; however, this observation should be interpreted cautiously given the small number of MRD-positive cases and the overall limited sample size. Finally, in our experience, Philadelphia chromosome status did not affect response rates to either monoclonal antibody.

It is less clear whether Blinatumomab and Inotuzumab can be used as standalone therapies. Consolidation by HCT had no impact on the OS of Blinatumomab-treated patients achieving CR or CRi in the TOWER trial (Kantarjian et al., 2017). On the other hand, Inotuzumab is typically used as a bridging therapy to allo-SCT, as remissions following Inotuzumab alone are short. Data from the INO-VATE ALL trial showed that the 2-year OS rate following Inotuzumab alone was 22.8%, inferior to that of patients bridged to allo-SCT (51%) (Marks et al., 2019).

It is thus clear that a subset of patients treated with immunotherapies can achieve durable remissions without allo-SCT. However, predictive biomarkers to identify these patients are lacking and it is thus reasonable to proceed to allo-SCT whenever possible, especially in Inotuzumab-treated cases. Data from our experience supports this supposition only partially. Two cases treated with Blinatumomab and DLI for post-transplant relapse showed remissions of 19 and 48 months respectively, with one patient alive, with CNS relapse at the date of study publication. With respect to Inotuzumab standalone treatment, in one patient with Ph positive disease and post-transplant relapse disease control was achieved by combining Inotuzumab and Dasatinib, with the patient alive in remission at the date of publication (42 months of remission, of which 24 TKI-free).

### The toxicities of immunotherapies

Furthermore, the selection of immunotherapy is influenced by the patient’s comorbidities and the expected toxicity profile of the two mAbs. CRS is encountered in roughly 4.9% of Blinatumomab treated patients, if the dose ramp-up schedule and pre-medication with Dexamethasone are followed (Kantarjian et al., 2017). Neurotoxicity occurs in about 9.3% of cases (Kantarjian et al., 2017; Maude et al., 2018), and a much higher incidence has been observed if Blinatumomab is administered for MRD positive disease (as high as 53%) (Kantarjian et al., 2017). De-bulking strategies can be thus considered before Blinatumomab administration to lower the incidence of CRS. Compared with literature data, we observed a higher incidence of CRS in our study group (19%). However, this complication was noticed exclusively in patients with a higher BM blast count (data not shown).

On the other hand, the most concerning toxicity related to Inotuzumab has been SOS. This complication has been evidenced in 14% of patients treated in the Inotuzumab arm, especially in those who relapsed after allo-SCT, or who were bridged to this procedure, especially if a dual-alkylator conditioning regimen was used. SOS war more frequent in patients receiving more than two cycles of Inotuzumab (41% vs. 17%) (Kantarjian et al., 2016). Thus, the following factors must be considered before using Inotuzumab: 1) pre-existent liver illness; 2) if a myeloablative conditioning regimen is likely to be used; 3) the avoidance of a dual-alkilator conditioning regimen; 4) the risk of excessive liver toxicity following SCT. Close collaboration with the SCT center is important to determine the optimal timing for allo-SCT and to limit the number of Inotuzumab cycles in transplant eligible patients. In our patient group, two cases developed SOS, of which one in a patient subjected to a conditioning regimen containing Melphalan and Thiothepa. The decision to pursue a dual alkylator conditioning regimen must be thus carefully weighed against the risk of SOS.

### The expression of the targeted antigen

CD19 is constantly expressed in B-ALL, but variations exist at relapse (Aldoss et al., 2017). The intensity of CD19 expression has not been correlated with Blinatumomab response but has been linked to peak CAR-T cell expansion, suggesting that higher levels of antigen expression are needed to harness the full potential of CAR-T cells (Curran and O’Brien, 2020; Maude et al., 2014). Thus, Blinatumomab might be preferred in cases with lower CD19 expression, as CAR-T cells are less able to respond to low levels of antigen (Curran and O’Brien, 2020).

CD22 is not uniformly expressed on B cell precursors and thus, patients must be screened for its expression by flow cytometry. The CD22 positivity threshold that predicts responsiveness to Inotuzumab is currently unknown. There are B-ALL subtypes, such as KMT2A-rearranged ALL, that have lower CD22 expression and poorer response to Inotuzumab, but it remains unclear whether this directly related to CD22 expression (Jabbour et al., 2019). In our study group, CD19 and CD22 expression was not correlated with the responses to Inotuzumab or Blinatumomab.

### The leukemic burden

In the TOWER trial, a lower percentage of BM blasts was associated with a higher CR rate (65% for patients with less than 50% BM blasts vs. 34.4% for a percentage above 50) (Kantarjian et al., 2017). The fact that Blinatumomab is more effective against a lower leukemic burden has also been highlighted by its improved effectiveness against MRD positive disease (Stein et al., 2019). Based on this data, a debulking strategy consisting of corticosteroids and/or chemotherapy should be considered to maximize the response to Blinatumomab. However, since Blinatumomab relies on the activation of autologous T cells, lymphodepleting agents should be avoided. On the other hand, the response and toxicity of Inotuzumab are not dependent on the leukemia burden (Kantarjian et al., 2016). Based on this data, Inotuzumab is an attractive option for patients with higher disease burden. In our study group, there was no correlation between the leukemic burden and the response rates between the two monoclonals.

### CNS and extramedullary leukemia

Blinatumomab and Inotuzumab do not cross the blood-brain barrier and are thus ineffective in this setting. Furthermore, extramedullary leukemia has been shown to predict Blinatumomab failure (Aldoss et al., 2017). CAR-T cells, on the other hand, can eradicate CNS disease, but are reserved for patients younger than 25 years. Alternative regimens for older patients include chemotherapy, either intrathecal or systemic, or radiotherapy. Since thiothepa, an alkilator agent, is often a part of conditioning regimens for B-ALL patients, especially with CNS disease, the benefit of using Inotuzumab as bridging treatment must be thoroughly weighed against the risk of later SOS. In our study group, CNS infiltration occurred in 9% of patients and was responsive to either IT followed by systemic cytarabine-based chemotherapy or to cerebrospinal irradiation. Overall, CNS involvement was successfully resolved in all cases and did not impact OS (*p* = 0.3, log-rank test) and DFS (*p* = 0.7, log-rank test).

### Patients treated sequentially with both mAbs

Patients who require sequential treatment with both monoclonals are heterogeneous. On one hand, patients who experience late relapses after having initially responded to the first immunotherapy (followed or not by alloSCT) have the potential to achieve a second CR. On the other hand, cases that are refractory to one of the monoclonals and require a second immunotherapy are particularly difficult to treat and have dismal outcomes. The response rate to the first monoclonal was clearly superior to the second (87.5% versus 37.5%) and was slightly higher for the Inotuzumab. Nonetheless, the small sample size precludes any firm conclusions regarding the superiority of one agent over the other. Survival after the second monoclonal was dismal, with 87.5% of patients dying within 1 month due to either disease progression (71.4%) or transplant-related complications (28.6%). Only one patient, who was bridged with Blinatumomab to CAR-T therapy, remains alive at the time of publication. This data indirectly suggests that, unless the second immunotherapy serves as a bridge to a potentially curative approach, the likelihood of response remains low. As such, its use may be questionable, particularly in real-life, underfunded healthcare settings, where its efficacy may not significantly exceed that of best supportive care.

### Post-alloSCT relapse and the effect of immunotherapy on the GVL effect and GVHD

There is a scarcity of data regarding the influence of post-ASCT immunotherapy on the GVL effect or the exacerbation of GVHD. Post-ASCT relapse in B-ALL has few therapeutic options, among which are the donor lymphocyte infusions (DLI), which rely on the graft versus leukemia (GVL) effect.

Theoretically, Blinatumomab promotes GVL by recruiting polyclonal T cells at relapse sites, and promote their differentiation in leukemia-specific memory and effector cells. In the case of co-treatment with DLIs and Blinatumomab, their administration should rather be synchronous, to maximize the opportunity for the formation of immune synapses. Indeed, T cell activation and the re-induction of full donor chimerism has been documented, in small patient groups, following Blinatumomab as either standalone therapy (Alcharakh et al., 2016) or in association with DLIs and tacrolimus in case of GHVD risk (Choi et al., 2020; Durer et al., 2019; Ueda et al., 2016). The reports of post-Blinatumomab GVHD are scarce and the mechanism, unclear. Since CD19 expression is absent in tissues targeted by GVHD, we can conclude that the indirect, immunomodulatory properties of Blinatumomab might be responsible for inducing it (Nägele et al., 2017; Khan and Gul, 2016). However, given the complex pathogenesis of GVHD, it is reasonable to hypothesize that in patients co-treated with Blinatumomab and DLIs, multiple factors contribute to GVHD development. This process cannot be attributed solely to the immunomodulatory effects of Blinatumomab and is likely driven predominantly by donor-derived immune cells (Frey and Porter, 2008).

Thus, Blinatumomab appears to be an effective and safe option for post-transplant relapse, and its combination with DLI and GVHD prophylaxis in patients at risk of GVHD holds promise and justifies further research.

Inotuzumab and DLIs seem to be a safe and promising combination in relapsed acute lymphoblastic leukemia after allogeneic stem cell transplant (Papayannidis et al., 2021) and in combination with Ponatinib in Ph positive B-ALL (Pirosa et al., 2018). Theoretically, the mechanism of action of Inotuzumab carries no risk of inducing GVHD and may offer an additional advantage by rapidly reducing the leukemic burden, thereby creating a more favorable context for the donor lymphocytes to eradicate residual tumor cells (Papayannidis et al., 2021).

The four post-transplant relapse cases described in our study may offer preliminary insight into the differential use of immunotherapies in this setting. Blinatumomab, which depends more on the functional capacity of donor-derived T cells and those infused via DLI, may be more appropriate for late relapses with lower disease burden. Conversely, in early, overt hematologic relapses, which occur under immune suppression and where the leukemic proliferation may outpace T cell activation and proliferation, Inotuzumab could represent a more suitable option.

### The subsequent need for allo-SCT or CAR-T cells

The subsequent need for allo-SCT or CAR-T cell therapy further impacts on the choice of immunotherapy. Allo-SCT relies on donor T cells eliminating residual leukemic cells through recognition of minor histocompatibility antigens and therefore requires achieving a deep therapeutic response, ideally CR with MRD negativity. In contrast, CAR-T cells require the presence of active leukemia to support their expansion, raising concerns that achieving an MRD-negative CR prior to infusion might impair CAR-T cell proliferation.

Inotuzumab induces complete responses in a significant percentage of patients, making it an attractive option for bridging to allo-SCT. However, the buildup of multiple alkylating agents in the conditioning regimen increases the risk of SOS. Virtually all patients are at risk of CNS relapse, so the addition of Thiotepa or total body irradiation is imperative and post-transplant cyclophosphamide is required in the prophylaxis of chronic GVHD for haploidentical or matched unrelated donor transplants. Therefore, while Inotuzumab is an appealing option for re-inducing remission, transplant physicians must remain aware of its broad impact on the conditioning and immune suppression strategies. On the other hand, the rate of CR and MRD negativity raise serious concerns about the use of Inotuzumab as bridging therapy to CAR-T, since the successful expansion of CAR-T relies on the existence of active leukemia.

Blinatumomab is a safer option for bridging to allo-SCT, but its efficacy seems to be lower than that of Inotuzumab, since it relies on each individual patient’s endogenous T cell fitness. Furthermore, the use of Blinatumomab as bridging to CAR-T should be done cautiously, as it can promote the emergence of CD19 negative subclones which lead to antigen negative relapses.

Therefore, given that both monoclonal antibodies have limitations when used as a bridge to CAR-T therapy and that CD22-directed CAR-T cells are not yet available, the choice between using one monoclonal agent over the other or opting for cytoreductive chemotherapy must be made on a case-by-case basis.

We acknowledge the limitations of our study: the retrospective design, the relatively small cohort size, the absence of multivariate analyses, and the heterogeneity of prior treatments, including exposure to agents that may impact the efficacy of subsequent immunotherapy. Despite these constraints, we believe our study adds valuable real-world evidence regarding the use and sequencing of Blinatumomab and Inotuzumab in adult R/R B-ALL.

## Conclusion

This retrospective, real-world study from two major oncology centers in Romania provides valuable insight into the use of Blinatumomab and Inotuzumab in the treatment of R/R B-ALL. Both agents demonstrated efficacy in achieving remission and MRD negativity, with Inotuzumab showing higher response rates overall, particularly in patients with higher leukemic burden or early relapse. Blinatumomab, on the other hand, may be better suited for patients with preserved T-cell function, lower disease burden, or late relapses, including those occurring post-transplant.

The optimal sequencing of the immuneotherapies remains a challenge. While initial responses to either agent can be robust, outcomes following a second monoclonal therapy are poor unless used as a bridge to curative interventions such as allo-SCT or CAR-T therapy. In such cases, thoughtful sequencing is critical, especially in the context of limited resources and potential toxicities. Furthermore, the integration of immunotherapy into post-transplant relapse management provides consistent results, especially in association with TKIs or DLI.

It is undoubtable that the therapeutic landscape of B-ALL is actively changing, as the novel immunotherapy agents are moving towards the first line of therapy. However, irrespectively of the therapeutic line they are used in, the presence of potential severe toxicity and the existence of a group of non-responders underlines the need for biomarkers predictive of response, so that these patients can be preemptively identified and spared the toxicity, especially in resource-constrained healthcare systems. It will be a challenge for future studies to define such biomarkers and integrate them into valid therapeutic strategies.

## Data Availability

The original contributions presented in the study are included in the article/Supplementary Material, further inquiries can be directed to the corresponding author.

## References

[B1] Alcharakh M. Yun S. Dong Y. Vincelette N. D. Daud M. Manzoor S. (2016). Blinatumomab-induced donor T-cell activation for post-stem cell transplant-relapsed acute CD19-positive biphenotypic leukemia. Immunotherapy 8 (8), 847–852. 10.2217/imt-2015-0023 27381683

[B2] AldossI. SongJ. StillerT. NguyenT. PalmerJ. O’DonnellM. (2017). Correlates of resistance and relapse during blinatumomab therapy for relapsed/refractory acute lymphoblastic leukemia. Am. J. Hematol. 92 (9), 858–865. 10.1002/ajh.24783 28494518

[B3] Algeri M. Massa M. Pagliara D. Bertaina V. Galaverna F. Pili I. (2025). Outcomes of children and young adults with B-cell acute lymphoblastic leukemia given blinatumomab as last consolidation treatment before allogeneic hematopoietic stem cell transplantation. Haematologica 110 (3), 596–607. 10.3324/haematol.2024.286350 39479863 PMC11873699

[B4] Badar T. Szabo A. Advani A. Wadleigh M. Arslan S. Khan M. A. (2020a). Real-world outcomes of adult B-cell acute lymphocytic leukemia patients treated with blinatumomab. Blood Adv. 4 (10), 2308–2316. 10.1182/bloodadvances.2019001381 32453836 PMC7252553

[B5] Badar T. Szabo A. Wadleigh M. Liedtke M. Arslan S. Siebenaller C. (2020b). Real-world outcomes of adult B-Cell acute lymphocytic leukemia patients treated with inotuzumab ozogamicin. Clin. Lymphoma Myeloma Leuk. 20 (8), 556–560.e2. 10.1016/j.clml.2020.03.004 32291234

[B6] Cairo M. S. Bishop M. (2004). Tumour lysis syndrome: new therapeutic strategies and classification. Br. J. Haematol. 127 (1), 3–11. 10.1111/j.1365-2141.2004.05094.x 15384972

[B7] Choi H. J. Choi J. Y. Kim B. K. An H. Y. Hong K. T. Shin H. Y. (2020). Combination therapy with chemotherapy, donor lymphocyte infusion with concurrent blinatumomab in relapsed/refractory acute precursor B-Lymphoblastic leukemia. J. Pediatr. Hematol. Oncol. 42 (7), e548–e553. 10.1097/MPH.0000000000001789 32251153

[B8] Curran E. O’Brien M. (2020). Role of blinatumomab, inotuzumab, and CAR T-cells: which to choose and how to sequence for patients with relapsed disease. Semin. Hematol. 57 (3), 157–163. 10.1053/j.seminhematol.2020.11.001 33256906

[B9] Durer S. Durer C. Shafqat M. Comba I. Y. Malik S. Faridi W. (2019). Concomitant use of blinatumomab and donor lymphocyte infusion for mixed-phenotype acute leukemia: a case report with literature review. Immunotherapy 11 (5), 373–378. 10.2217/imt-2018-0104 30786841 PMC6439498

[B10] Frey N. V. Porter D. L. (2008). Graft-versus-host disease after donor leukocyte infusions: presentation and management. Best. Pract. Res. Clin. Haematol. 21 (2), 205–222. 10.1016/j.beha.2008.02.007 18503987 PMC2504712

[B11] Gökbuget N. Stanze D. Beck J. Diedrich H. Horst H. A. Hüttmann A. (2012). Outcome of relapsed adult lymphoblastic leukemia depends on response to salvage chemotherapy, prognostic factors, and performance of stem cell transplantation. Blood 120 (10), 2032–2041. 10.1182/blood-2011-12-399287 22493293

[B12] Hodder A. Mishra A. K. Enshaei A. Baird S. Elbeshlawi I. Bonney D. (2024). Blinatumomab for first-line treatment of children and young persons with B-ALL. J. Clin. Oncol. 42 (8), 907–914. 10.1200/JCO.23.01392 37967307

[B13] Jabbour E. Advani A. S. Stelljes M. Stock W. Liedtke M. Gökbuget N. (2019). Prognostic implications of cytogenetics in adults with acute lymphoblastic leukemia treated with inotuzumab ozogamicin. Am. J. Hematol. 94 (4), 408–416. 10.1002/ajh.25394 30623490

[B14] Jabbour E. Gökbuget N. Advani A. Stelljes M. Stock W. Liedtke M. (2020). Impact of minimal residual disease status in patients with relapsed/refractory acute lymphoblastic leukemia treated with inotuzumab ozogamicin in the phase III INO-VATE trial. Leuk. Res. 88, 106283. 10.1016/j.leukres.2019.106283 31790983

[B15] Jabbour E. J. Rousselot P. Gokbuget N. Chevallier P. Kantarjian H. M. Stelljes M. (2025). Inotuzumab ozogamicin as first-line therapy in acute lymphoblastic leukemia. Clin. Lymphoma Myeloma Leuk. 25 (5), e302–e309. 10.1016/j.clml.2024.12.016 39909815

[B16] Kantarjian H. M. O’Brien S. Smith T. L. Cortes J. Giles F. J. Beran M. (2000). Results of treatment with hyper-CVAD, a dose-intensive regimen, in adult acute lymphocytic leukemia. J. Clin. Oncol. 18 (3), 547–561. 10.1200/JCO.2000.18.3.547 10653870

[B17] Kantarjian H. M. DeAngelo D. J. Stelljes M. Martinelli G. Liedtke M. Stock W. (2016). Notuzumab ozogamicin Versus standard care for acute lymphoblastic leukemia. N. Engl. J. Med. 375 (8), 740–753. 10.1056/NEJMoa1509277 27292104 PMC5594743

[B18] Kantarjian H. Stein A. Gökbuget N. Fielding A. K. Schuh A. C. Ribera J. M. (2017). Blinatumomab versus chemotherapy for advanced acute lymphoblastic leukemia. N. Engl. J. Med. 376 (9), 836–847. 10.1056/NEJMoa1609783 28249141 PMC5881572

[B19] Khan M. W. Gul Z. (2016). Blinatumomab may induce graft versus host leukemia in patients with pre-B ALL relapsing after hematopoietic stem cell transplant. Clin. Case Rep. 4 (8), 743–746. 10.1002/ccr3.604 27525074 PMC4974418

[B20] Khazal S. Kebriaei P. (2020). Debate: transplant is still necessary in the era of targeted cellular therapy for acute lymphoblastic leukemia. Clin. Lymphoma, Myeloma Leuk. 20 (11), 713–719. 10.1016/j.clml.2020.06.011 32694050

[B21] Marks D. I. Kebriaei P. Stelljes M. Gökbuget N. Kantarjian H. Advani A. S. (2019). Outcomes of allogeneic stem cell transplantation after Inotuzumab ozogamicin treatment for relapsed or refractory acute lymphoblastic leukemia. Biol. Blood Marrow Transpl. 25 (9), 1720–1729. 10.1016/j.bbmt.2019.04.020 31039409

[B22] Maude S. L. Frey N. Shaw P. A. Aplenc R. Barrett D. M. Bunin N. J. (2014). Chimeric antigen receptor T cells for sustained remissions in leukemia. N. Engl. J. Med. 371 (16), 1507–1517. 10.1056/NEJMoa1407222 25317870 PMC4267531

[B23] Maude S. L. Laetsch T. W. Buechner J. Rives S. Boyer M. Bittencourt H. (2018). Tisagenlecleucel in children and young adults with B-Cell lymphoblastic leukemia. N. Engl. J. Med. 378 (5), 439–448. 10.1056/NEJMoa1709866 29385370 PMC5996391

[B24] Nägele V. Kratzer A. Zugmaier G. Holland C. Hijazi Y. Topp M. S. (2017). Changes in clinical laboratory parameters and pharmacodynamic markers in response to blinatumomab treatment of patients with relapsed/refractory ALL. Exp. Hematol. Oncol. 6 (1), 14. 10.1186/s40164-017-0074-5 28533941 PMC5437652

[B25] Oriol A. Vives S. Hernández-Rivas J. M. Tormo M. Heras I. Rivas C. (2010). Outcome after relapse of acute lymphoblastic leukemia in adult patients included in four consecutive risk-adapted trials by the PETHEMA study group. Haematologica 95 (4), 589–596. 10.3324/haematol.2009.014274 20145276 PMC2857188

[B26] Papayannidis C. Sartor C. Dominietto A. Zappone E. Arpinati M. Marconi G. (2021). Inotuzumab ozogamicin and donor lymphocyte infusion is a safe and promising combination in relapsed acute lymphoblastic leukemia after allogeneic stem cell transplant. Hematol. Oncol. 39 (4), 580–583. 10.1002/hon.2886 33963566

[B27] Petre I. Barna F. Gurgus D. Tomescu L. C. Apostol A. Petre I. (2023). Analysis of the healthcare system in Romania: a brief review. Healthc. (Basel) 11 (14), 2069. 10.3390/healthcare11142069 37510510 PMC10379121

[B28] Pirosa M. C. Leotta S. Cupri A. Stella S. Martino E. A. Scalise L. (2018). Long-term molecular remission achieved by antibody Anti-CD22 and ponatinib in a patient affected by Ph’+ acute lymphoblastic leukemia relapsed after second allogeneic hematopoietic stem cell transplantation: a case report. Chemotherapy 63 (4), 220–224. 10.1159/000492941 30372691

[B29] Proskorovsky I. Su Y. Fahrbach K. Vandendries E. Pagé V. Onyekwere U. (2019). Indirect treatment comparison of inotuzumab ozogamicin Versus blinatumomab for relapsed or refractory acute lymphoblastic leukemia. Adv. Ther. 36 (8), 2147–2160. 10.1007/s12325-019-00991-w 31140123 PMC6822860

[B30] Proskorovsky I. Vandendries E. Pagé V. Cappelleri J. C. Stelljes M. (2020). Response to letter to the editor regarding: indirect treatment comparison of inotuzumab ozogamicin *versus* blinatumomab for relapsed or refractory acute lymphoblastic leukemia. Adv. Ther. 37 (3), 958–962. 10.1007/s12325-019-01169-0 31838711 PMC7004421

[B31] Sayyed A. Chen C. Gerbitz A. Kim D. D. H. Kumar R. Lam W. (2024). Pretransplant blinatumomab improves outcomes in B cell acute lymphoblastic leukemia patients who undergo allogeneic hematopoietic cell transplantation. Transpl. Cell Ther. 30 (5), 520.e1–520.e12. 10.1016/j.jtct.2024.03.004 38462215

[B32] Song J. Ma Q. Gao W. Cong Z. Xie J. Zimmerman Z. (2019). Matching-adjusted indirect comparison of blinatumomab vs. inotuzumab ozogamicin for adults with relapsed/refractory acute lymphoblastic leukemia. Adv. Ther. 36 (4), 950–961. 10.1007/s12325-019-0873-7 30758745 PMC6824351

[B33] Song J. Gao W. Xie J. Tiwana S. (2020). Letter to the editor regarding: indirect treatment comparison of inotuzumab ozogamicin Versus blinatumomab for relapsed or refractory acute lymphoblastic leukemia. Adv. Ther. 37 (2), 955–957. 10.1007/s12325-019-01168-1 31838710 PMC6999160

[B34] Spyridonidis A. Labopin M. Schmid C. Volin L. Yakoub-Agha I. Stadler M. (2012). Outcomes and prognostic factors of adults with acute lymphoblastic leukemia who relapse after allogeneic hematopoietic cell transplantation. An analysis on behalf of the acute leukemia working party of EBMT. Leukemia 26 (6), 1211–1217. 10.1038/leu.2011.351 22290066

[B35] Stein A. S. Kantarjian H. Gökbuget N. Bargou R. Litzow M. R. Rambaldi A. (2019). Blinatumomab for acute lymphoblastic leukemia relapse after allogeneic hematopoietic stem cell transplantation. Biol. Blood Marrow Transpl. 25 (8), 1498–1504. 10.1016/j.bbmt.2019.04.010 31002989

[B36] Ueda M. De Lima M. Caimi P. Tomlinson B. Little J. Creger R. (2016). Concurrent blinatumomab and donor lymphocyte infusions for treatment of relapsed pre-B-cell ALL after allogeneic hematopoietic cell transplant. Bone Marrow Transpl. 51 (9), 1253–1255. 10.1038/bmt.2016.104 27088374

[B37] Vladescu C. Silvia V. Scîntee G. Olsavszky V. Hernández-Quevedo C. Sagan A. (2016). Romania: health system review. Health Syst. Transit 18 (4), 1–170. 27603897

[B38] Yoon J. H. Min G. J. Park S. S. Park S. Lee S. E. Cho B. S. (2019). Feasible outcome of blinatumomab followed by allogeneic hematopoietic cell transplantation for adults with Philadelphia chromosome-negative acute lymphoblastic leukemia in first salvage. Cancer Med. 8 (18), 7650–7659. 10.1002/cam4.2680 31691536 PMC6912052

