# Supplementary material for: Real-world patient outcomes with Blinatumomab and Inotuzumab in adult relapsed/refractory B-cell acute lymphoblastic leukemia: a retrospective analysis from two Romanian oncology centers
Source: Front Pharmacol. 2025 Dec 10;16:1684382. doi: 10.3389/fphar.2025.1684382 (PMC12728000; doi:10.3389/fphar.2025.1684382)
Supplement: Supplementary file 1 [file Table1.docx]

**Supplementary Table 1.** Previous therapies of patients treated with Blinatumomab.

| Therapies before Blinatumomab | Total patients treated with Blinatumomab (n=21), %, (n) |
| --- | --- |
| PETHEMA-ALL ‘93 | 28.5% (n=6) |
| Hyper-CVAD | 19% (n=4) |
| FLAG-IDA | 14.2% (n=3) |
| Allo-SCT | 9.5% (n=2) |
| Inotuzumab | 19% (n=4) |
| Other | 9.5% (n=2) |

Allo-SCT= allogeneic stem cell transplant, FLAG-IDA= Fludarabine, Cytarabine, Granulocyte Colony Stimulating Factor, Idarubicine, Hyper-CVAD= hyperfractionated Cyclophosphamide, Vincristine, Doxorubicin, Dexamethasone, PETHEMA-ALL= Programa Español de Tratamientos en Hematología- Acute Lymphoblastic Leukemia.
